# The Rate and Tract Length of Gene Conversion between Duplicated Genes

**DOI:** 10.3390/genes2020313

**Published:** 2011-03-25

**Authors:** Sayaka P. Mansai, Tomoyuki Kado, Hideki Innan

**Affiliations:** 1 Graduate University for Advanced Studies, Hayama, Kanagawa 240-0193, Japan; E-Mails: mansai_sayaka@soken.ac.jp (S.P.M.); kado_tomoyuki@soken.ac.jp (T.K.); 2 PRESTO, Japan Science and Technology Agency (JST), Saitama, 332-0012, Japan

**Keywords:** interlocus gene conversion, tract length, gene conversion rate

## Abstract

Interlocus gene conversion occurs such that a certain length of DNA fragment is non-reciprocally transferred (copied and pasted) between paralogous regions. To understand the rate and tract length of gene conversion, there are two major approaches. One is based on mutation-accumulation experiments, and the other uses natural DNA sequence variation. In this review, we overview the two major approaches and discuss their advantages and disadvantages. In addition, to demonstrate the importance of statistical analysis of empirical and evolutionary data for estimating tract length, we apply a maximum likelihood method to several data sets.

## 1. Introduction

Gene conversion is a recombinational process initiated by a double strand break (DSB), through which a DNA fragment is non-reciprocally transferred (copied and pasted) generally between allelic regions (*i.e.*, allelic gene conversion [1]). In addition, gene conversion occurs between paralogs when they have sufficient sequence homology (non-allelic or interlocus gene conversion) [2,3]. Although the mechanism is not fully understood yet (see Hastings [4] and Ling *et al.* [5] in this special issue), interlocus gene conversion is a major mutational process that occurs in both meiosis [2] and mitosis [6]. Basic questions on gene conversion include (i) What is the rate of gene conversion under what condition? and (ii) What is the distribution of the tract length of gene conversion? Addressing these fundamental questions will provide great insights into how important role gene conversion plays as a mutational mechanism.

There are two potential approaches to estimate the rate and tract length of gene conversion. A straightforward empirical approach involves mutation (*i.e.*, gene conversion) accumulation studies, in which the rate of gene conversion can be directly estimated. The other is an evolutionary approach, which utilizes DNA sequence data from multiple individuals, where the footprints of a number of gene conversion events are accumulated in the evolutionary history. The empirical approach provides more accurate estimates than the evolutionary approach, but the amounts of data are still limited because well-established experimental systems are available only for several model species including yeast and mouse. In contrast, the evolutionary approach can be readily applied to any species when sequence data for paralogs are available.

In this article, we first review researches that estimated the rate and tract length of interlocus gene conversion by the two approaches, and discuss their advantages and disadvantages. Furthermore, we reanalyze empirical data from yeast and rodent to estimate the mean tract length by using a simple maximum likelihood (ML) method. We also apply the ML method to large-scale human genotyping data of diseases genes, in which gene conversion is known to cause diseases. Based on these results, we point out the importance of statistical analysis of empirical and evolutionary data.

## 2. The Rate of Gene Conversion

### 2.1. Empirical Approach

**Figure 1:**
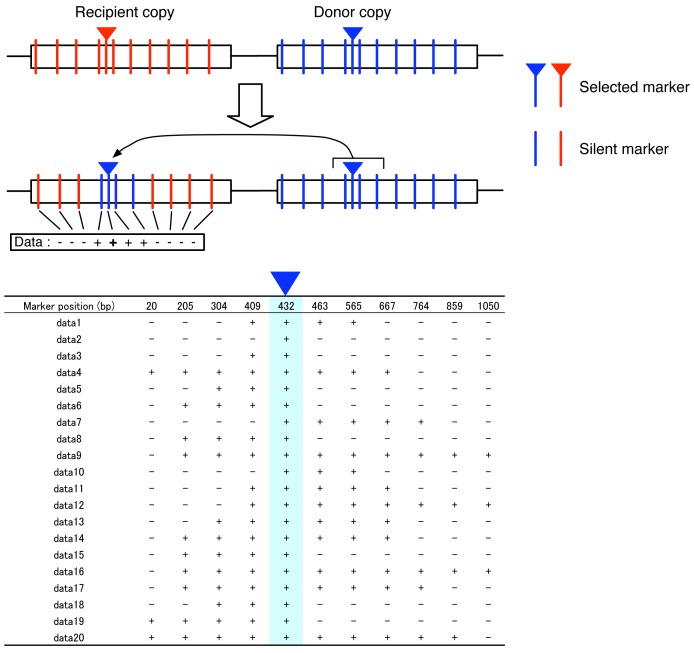
Illustration of a typical experiment to screen for gene conversion. See text for details.

Most transgenic systems for studying gene conversion use strains (or cell lines), in which a pair of genes are set up by transferring artificially edited DNA sequences (Figure 1), so that the nature of interlocus gene conversion can be investigated under an arbitrary condition [7]. The gene has a target marker site called “selected marker” (reversed triangles in Figure 1), and there is a trick that makes it possible to recognize if the selected marker is converted (e.g., in yeast, if a gene involved in nutrient requirement such as uracil or histidine is used, gene conversion induces prototroph formation). Therefore, it is easy to screen for strains that experienced gene conversions. Although Figure 1 illustrates a simple case, it is possible to set multiple selected markers.

In the baker's yeast *Saccharomyces cerevisiae*, one of the model species of gene conversion studies, there are a number of researches that estimated the interlocus gene conversion rate in various conditions [8–15]. Estimates have a wide range from ∼10^−10^ to ∼10^−3^ per cell division (we exclude classic works that reported estimates per culture). This great amount of variation between different researches largely depends on the fact that the gene conversion rate is affected by many factors.

First of all, the rate in meiosis is different from that in mitosis. Jinks-Robertson and Petes [12] showed that the former is 300 times higher than the latter, and slightly lower estimates for the meiosis-mitosis ratio were reported later (∼15 in [16] and ∼100 in [17]).

Second, genomic location matters. In general, the rate of intrachromosomal gene conversion may be higher than that of interchromosomal gene conversion. Lichten *et al.* [13,15] showed that the former is several times higher than the latter in both meiosis and mitosis (see also [14,18]). Lichten *et al.* [15] also investigated the positional effect on the gene conversion rate when two paralogs are located with variable distances between them. It was found that the gene conversion rate could be negatively correlated with the distance (see also [11]). It seems that the pattern may not be very different between in meiosis and in mitosis [13,15].

Third, the rate should be in a negative correlation with the sequence identity between paralogs. It is considered that gene conversion mainly occurs when the identity is more than 80% [19,20], but gene conversion can occur with identity <80% although the rate is low [21]. Recent works clearly identified a negative correlation between the sequence identity and gene conversion rate [22–25].

Fourth, it seems that the length of completely identical region has a significant effect on the rate of gene conversion. Ahn *et al.* [26] measured the rate with variable lengths of a recipient copy (from 26 bp to 702 bp), and found that although the rate was extremely low, gene conversion occurred even with the shortest paralog (*i.e.*, 26 bp), suggesting gene conversion requires identical regions as small as ∼10 bp (see also Mezard *et al.* [21] for a similar result). A later work by Jinks-Robertson and Petes [14] found that the rates for paralogs that have identical regions with >250 bp were much higher than the background rate. This work brought the concept of the minimal efficient processing segment (MEPS), which was first introduced for homologous recombination in bacteria [27]. Jinks-Robertson and Petes [14] suggested that MEPS for yeast would be around 200 bp, which still serves as a good standard [7,28], but this does not necessarily rule out the possibility of gene conversion with paralogs that are shorter than MEPS [21,23,26,29,30].

The empirical approach has also been commonly applied to mouse (*Mus musculus*) and Chinese hamster (*Cricetulus griseus*). Extensive experiments exhibited quite similar results to those of yeast. It seems that the rate in meiosis may be 100∼1000 times higher than in mitosis [31]. A negative correlation between the gene conversion rate and paralogous distance was observed [32,33]. It was found that the rate is dramatically reduced for paralogs with identity ∼80% in comparison with the rate for 100% identical pairs [34–36]. MEPS was estimated to be roughly 200 bp [35], which was repeatedly confirmed by follow-up experiments [37,38].

Thus, we overviewed experimental studies of yeast and rodent which explored the factors that affect the rate of gene conversion. These findings should be robust because each of them was demonstrated under a certain experimental condition. However, we found that it is difficult to compare the absolute gene conversion rates between different experiments, and this is why we avoided arguments with absolute values. For example, some researches take advantage of specific sites where DSBs can be induced, (e.g., the HO site in the *MAT* locus [39] and target site of I-*Sce* I endonuclease [40]) and some did not. A technical problem is that, in a simple experimental design with a single selected marker, an estimated rate may include both gene conversion and unequal crossing-over because they have an identical outcome. Such a rate cannot be fairly compared with an estimate from a more sophisticated experiment, in which the two mechanisms can be distinguished, e.g., by using secondary markers. Furthermore, *in vivo*, the rate should largely depend on the genomic background, for example, the number of paralogs in the genome [11], special motives associated with recombination [41,42], activity of numerous enzymes involved in DNA repair and recombination (reviewed in [43]). Therefore, it is important to notice that the available estimates may not be representative values of the gene conversion rate.

### 2.2. Evolutionary Approach

The pattern of polymorphism e.g., SNP in duplicated regions is very informative because it should have many footprints of gene conversion. This approach heavily depends on theoretical understanding of population genetics on what kind of polymorphism pattern is likely expected under what rate of gene conversion. When the process of gene conversion is modeled in the framework of population genetics, it is usually assumed that a gene conversion event can be initiated at a random position at a certain rate, *g*, and the elongation of the gene conversion tract occurs either in the 5′ or 3′ direction. It is commonly assumed that the elongation can be terminated at any position with a constant probability, say *q*, such that the tract length follows a geometric distribution with parameter *q*, or an exponential distribution with continuous approximation [44]. As the average tract length is *T* = 1/*q*, the per-site rate of gene conversion is defined as *c* = *Tg*, which is the probability that a particular site is involved in a gene conversion event per generation. According to population genetic theory [45–47], *c* can be well estimated from SNP data. From genome-wide SNP data in yeast [48], estimates of the relative rate of gene conversion *c* to the point mutation rate typically ranges from 10 to 100, which is in agreement with estimates for several duplicates in *Drosophila melaogaster* [49,50]. There are many locus-specific estimates of c for a wide range of species including malaria parasite [51], plant [52], avian [53] and human [54–56].

A disadvantage of this approach is that estimates are model-dependent; therefore, they could be biased if the assumptions of the model do not hold. For example, as Innan's theory [45,46] assumes a constant-size population, an estimate may not be reliable if the population is growing or subdivided. This is a common problem shared by all estimates by population genetics-based methods, such as those of mutation rate and recombination rate. It is important to understand how population genetics-based estimates are quantitatively affected by violation of the assumptions. Coalescent simulations [46] would be one of the best ways to do this.

## 3. The Tract Length of Gene Conversion

### 3.1. Empirical Approach

It is relatively straightforward to develop an empirical system for estimating gene conversion tract length by modifying the transgenic system for estimating the gene conversion rate. Figure 1 illustrates such a strategy, which has a selected marker at position 432 bp. In addition to the selected marker, there are several silent markers inserted in the donor gene. Because silent markers could be coconverted with the selected marker, it is possible to identify the converted tract in the marker space. Figure 1 also illustrates an example of a gene conversion event, which includes four markers from positions 409 to 565. It can be inferred that the 5′ break point should be between positions 304 and 409 and the 3′ break point should locate between positions 565 and 667. Therefore, the maximum and minimum lengths of this conversion tract are 362 bp and 157 bp, respectively. Thus, the data of a number of detected gene conversion can be described by a simple matrix with the presence(+)/absence(−) of the markers in the recipient copy (Figure 1). Note that all data should have a positive sign (+) at the selected marker with the blue triangle.

Motivated by classic works [57,58], Ahn *et al.* [59] extensively investigated the tract length of gene conversion in yeast, and found the average of the minimum lengths is about 500 bp. There are a number of follow-up studies, most of which reported an estimate of the average of 200–300 bp [60–64] (Note that most of them reported the mean of the maximum and minimum lengths). It seems that there is no big difference in the tract length of gene conversion between mitosis and meiosis [25]. Similar estimates (287–296 bp) have also been obtained for rodents (Chinese hamster) [63,64].

**Figure 2:**
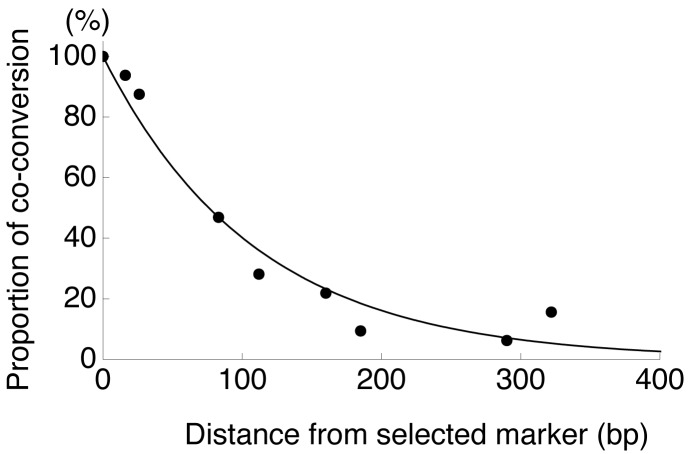
The proportion of coconverted silent markers as a function of distance from the selected marker. Data from Taghian *et al.* [63].

Thus, this experimental system has contributed to our understanding of the typical length of gene conversion. However, a problem is that the initiation and termination positions of the tract are usually identified between markers, and it is difficult to know the actual length. It is more serious when a tract covers all markers so that the tract is potentially very long. Therefore, to understand the distribution of actual tract length, it is needed to analyze the data statistically [65–69]. Here, we use a maximum likelihood (ML) method to estimate the mean tract length by assuming that the elongation of a gene conversion tract is terminated at a constant rate. This assumption should be reasonable because we found that the data of Taghian *et al.* [63] and others [59,61] exhibit a good fit to a geometric distribution, or an exponential distribution with continuous approximation (Figure 2).

**Figure 3:**
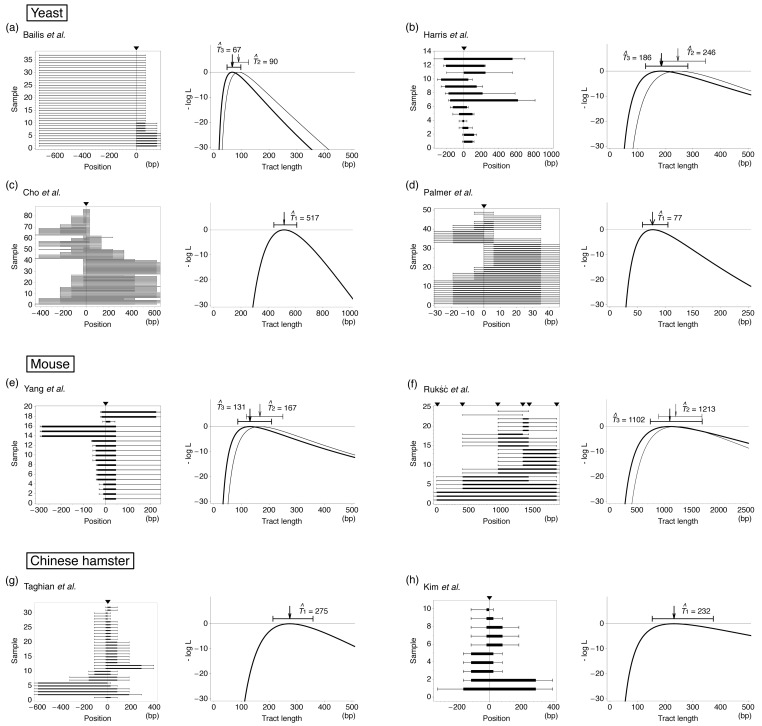
Application of the ML method to the eight data sets in Table 1. For each data set, the minimum and maximum lengths of identified fragments are shown in the left panel. The minimum length is given by a filled box with the maximum length by a bar. Filled triangles indicate the positions of the selected markers. The right panel shows the log-likelihood curve and the ML estimate *T̂* with the 95% confidence interval.

Table 1 summarizes data which are suitable to infer the mean tract length of interlocus gene conversion with our ML method. We collected data sets with the number of markers *M* ≥ 4 and the sample size *n* ≥ 10. All data in Table 1 are for mitosis. For each detected gene conversion, the maximum and minimum lengths can be parsimoniously determined and summarized in Figure 3. We excluded data that need double conversion events to explain parsimoniously. Such data (called discontinuous tracts) arise at a low frequency [19,59–63]. Figure 3 shows that many tracts involve only the selected marker, indicting there are a large number of short gene conversion. On one hand, there are many tracts that could be potentially very long because they have positive signs at the left- and/or right-end markers.

**Table 1.** Summary of the data used for the ML analysis.

**Data Set**	**Donor / Recipient Genes**^a^	**Length of Analyzed Region**	**Sequence Identity**	***M* (# of Markers)**	***n* (Sample Size)**	**Gene Conversion Rate**^b^
***Saccharomyces cerevisiae* (yeast)**						
Bailis *et al.* [19]	*SAM2* (*IV*) / *SAM1* (*XII*)	869 bp	83%	4	37	8.4 × 10^−9^
Harris *et al.*[20]	*PMA1* (*VII*) / *PMA2* (*XVI*)	1830 bp	85%	24	13	5.0 × 10^−9^
Cho *et al.* [61]	*ura3* / *ura3* (4.9 kb)	1130 bp	99%	11	86	1.6 × 10^−5^ (3.8 × 10^−3^) ^c^
Palmer *et al.* [70]	*ura3* / *ura3* (4.9 kb)	54 bp	99%	5	49	3.3 × 10^−6^ (1.1 × 10^−3^) c

***Mus musculus* (mouse)**						
Yang *et al.* [71]	HSV-1 *tk* / -2 *tk* (<6.5 kb)	2500 bp	81%	14, 8^d^	19	1.3 × 10^−8^ (2.3 × 10^−7^)
Rukść *et al.* [72]	*Cμ*5′ / *Cμ*3′ (<8 kb)	1876 bp	99%	6	24	n.d.

***Cricetulus griseus* (Chinese hamster)**						
Taghian *et al.* [63]	MMTV*neo* / *neo12* (3.8 kb)	989 bp	99%	13	32	< 4.8 × 10^−10^ (2.7 × 10^−5^) e
Kim *et al.* [64]	MMTV*neo* / *neo12* (3.8 kb)	989 bp	99%	10	11	< 5.6 × 10^−10^ (2.3 × 10^−5^) f

a The chromosome numbers of the donor and recipient genes are shown in the parentheses if they are located on different chromosomes. If they are on the same chromosome, the distance between them is shown.

b Estimated rate from a strain with accelerated DSB is shown in parentheses.

c Computed by assuming the doubling time is 3 h and the culturing time is 3 days.

d We pooled two data sets with different numbers of markers, 14 and 8.

e Computed by assuming the doubling time is 15 h and the culturing time is14 days.

f Computed by assuming the doubling time is 15 h and the culturing time is 12 days.

In order to estimate the average length of gene conversion, *T*, from these data, we use a simple ML method. Our method is a slightly modified version of Gloor *et al.* [65] (see also [66]) so that it is possible to apply to data with multiple selected markers. Let **D** be the data set obtained from a single pair of genes, which consists of *n* identified tracts. Each tract is characterized by (*i*, *j*) when the tract includes a region from the *i*th to *j*th markers (*i* ≤ *j*) but not *i*−1 or *j*+1th markers. Note that *i* ≤ *k* ≤ *j* when the *k*th marker is the selected marker that is used for detecting gene conversion. Then, **D** can be simply described as

$$\text{D}=\left\{\left(i_{1},\;j_{1}\right),\;\left(i_{2},\;j_{2}\right),\;\cdots ,\left(i_{n},\;j_{n}\right)\right\}$$

where (*i_l_*, *j_l_*) is for the *l*th tract (*l* = 1, 2, 3, …, *n*). *M* represents the total number of markers, and the position of the *i*th marker is denoted by *m_i_* (*i* = 1, 2, 3, …, *M*). In addition, we define *m_0_* = *L*_−_ and *m_M_*_+1_ = *L*_+_, where *L*_−_ and *L*_+_ are the left and right ends of the duplicated region. We here assume *L*_−_ ≪ *m*_1_ and *L*_+_ ≫ *m_M_* (*i.e.*, *L*_−_ = −∞ and *L*_+_ = ∞ for mathematical convenience), which makes the following computation much easier with a negligible effect on the ML estimate.

To obtain the likelihood function of data **D** conditional on *T*, we consider three different models depending on the experimental design and the distribution of gene conversion tract. The first model (Model 1) can be applied to many experimental designs, in which DSBs are induced at a particular position (denoted by *x_k_*) and the selected marker is essentially identical to the induced position. In such a case, it is straightforward to assume that the elongation of converted tracts in the two directions independently follow an exponential distribution (see above and Figure 2). Let *x*_1_ and *x*_2_ be the 5′ and 3′ breakpoints of the tract. Then, the probability that a tract that includes the selected marker is from position *x*_1_ to *x*_2_ is given by a function of *T*:

(1) $$\begin{array}{cc} p_{1}(x_{1},\;x_{2}\;|\;T) & =\frac{\frac{2}{T}e^{-\frac{2\left(x_{k}-x_{1}\right)}{T}}\times \frac{2}{T}e^{-\frac{2\left(x_{2}-x_{k}\right)}{T}}}{\int_{L_{-}}^{x_{k}}\frac{2}{T}e^{-\frac{2\left(x_{k}-x_{1}\right)}{T}}dx_{1}\times \int_{x_{k}}^{L_{+}}\frac{2}{T}e^{-\frac{2\left(x_{2}-x_{k}\right)}{T}}dx_{2}}\\ & =\frac{4}{T^{2}}e^{-\frac{2\left(x_{2}-x_{1}\right)}{T}} \end{array}$$

Then, because the focal tract has to satisfy the two conditions, *m_i_*_−1_ < *x*_1_ < *m_i_* and *m_j_* < *x*_2_< *m_j_*_+1_, we have the probability that a conversion tract is given by (*i*, *j*):

(2) $${\text{Prob}}_{1}\left(i,\;j\;|\;T\right)=\int_{m_{i-1}}^{m_{i}}\int_{m_{j}}^{m_{j+1}}p_{1}\left(x_{1},\;x_{2}\;|\;T\right)dx_{2}dx_{1}$$

Then, the likelihood of the data **D** is given by

(3) $$L_{1}\left(T\;|\;\text{D}\right)=\prod_{l=1}^{n}{\text{Prob}}_{1}(i_{l},\;j_{l}\;|\;T).$$

In other cases, the location of DSBs should be treated to be unknown. If we assume the exponential elongation of a tract from a DSB in the two directions independently as defined in (1), then the total length follows a gamma distribution with shape parameter two. In this Model 2, we assume this gamma distribution for the length of an individual tract (*t*):

(4) $$p_{2}\left(t\right)=\frac{4t}{T^{2}}e^{-\frac{2t}{T}}$$

and *Prob*_2_(*i*, *j*∣*T*), the probability that a conversion tract is given by (*i*, *j*) conditional on a conversion event that involves at least one markers is given by

(5) $${\text{Prob}}_{2}\left(i,\;j\;|\;T\right)=\frac{\int_{m_{i-1}}^{m_{i}}\int_{m_{j}}^{m_{j+1}}p_{2}\left(x_{2}-x_{1}\right)dx_{2}dx_{1}}{\sum_{\left(i,j\right)\subset \text{A}}\int_{m_{i-1}}^{m_{i}}\int_{m_{j}}^{m_{j+1}}p_{2}\left(x_{2}-x_{1}\right)dx_{2}dx_{1}}$$

where **A** consists of all possible pairs of (*i*, *j*) that satisfy the condition, *i* ≤ *j*.

From (5), we can obtain the probability that a gene conversion event is captured in the experimental system. In other words, we are interested in the probability that the tract includes the selected marker (*i.e.*, *k*th marker). Assume that **B** consists of all possible pairs of (*i*, *j*) that satisfy the condition, *i* ≤ *k* ≤ *j*. Then, this probability is given by

(6) $${\text{Prob}}_{2}\left(\text{B}\;|\;T\right)=\sum_{\left(i,j\right)\subset \text{B}}{\text{Prob}}_{2}\left(i,\;j\;|\;T\right)$$

From (5) and (6), we have the probability that the detected tract is (*i*, *j*):

(7) $${\text{Prob}}_{2}\left(i,\;j\;|\;\text{B},T\right)=\frac{{\text{Prob}}_{2}\left(i,\;j\;|\;T\right)}{{\text{Prob}}_{2}\left(\text{B}\;|\;T\right)}$$

and the likelihood of the entire data **D** is given by

(8) $$L_{2}\left(T\;|\;\text{D}\right)=\prod_{l=1}^{n}\frac{{\text{prob}}_{2}\left(i_{l},\;j_{l}\;|\;T\right)}{{\text{prob}}_{2}\left(\text{B}\;|\;T\right)}$$

In addition, we consider Model 3, in which the entire length of a tract follows an exponential distribution:

(9) $$p_{3}\left(t\right)=\frac{1}{T}e^{-\frac{t}{T}}$$

This is a frequently used assumption in evolutionary models as introduced in the “Evolutionary approach” section. For this Model 3, the likelihood function of **D** is simply given by (5) by replacing *p*_2_(*t*) with *p*_3_(*t*).

Using these likelihood functions, we estimated *T* for each data set in Table 1 and the results are shown in Figure 3. If the data set has a selected marker at the position of induced DSB, Model 1 is applied, otherwise both Models 2 and 3 are used. ML estimates based on these three models are denoted by *T̂*_1_, *T̂*_2_ and *T̂*_3_. It was found that our method provides ML estimates of the mean tract length with relatively narrow confidence interval. The results of Models 2 and 3 are not very different to each other. For the four data sets of yeast, the estimates are within a quite small range from ∼50 to several hundred bp, which seems to be much smaller than allelic gene conversion [73]. The results for rodents are similar; our estimates are around 100 bp except for the data of Rukść *et al.* [72], which provide an estimate of >1 kb with a much wider confidence interval (roughly 1 kp) than the others. This may be partly because the marker density is very low (the average interval is 375 bp for this data set, while the average of the others is ∼160 bp).

Thus, the ML method makes it possible to estimate the mean tract length with relatively small amount of data. It is demonstrated that the empirical approach with statistical analyses is a powerful means to understand the tract length of gene conversion.

### 3.2. Evolutionary Approach

DNA sequence data potentially include information on the tract lengths of gene conversion events that occurred in their ancestral lineages. GENECONV is a software developed by Sawyer [74] to detect converted regions in aligned DNA sequences. GENECONV analyzes an alignment of multiple sequences in a pairwise manner, and identifies unusually long regions of high identity between the focal pair, which are candidates of gene conversion. The algorithm involves statistical treatment, which conditions on the pattern of variable sites in the other sequences in the alignment. The statistical significance is determined by random-shuffling of variable sites in the alignment.

**Figure 4:**
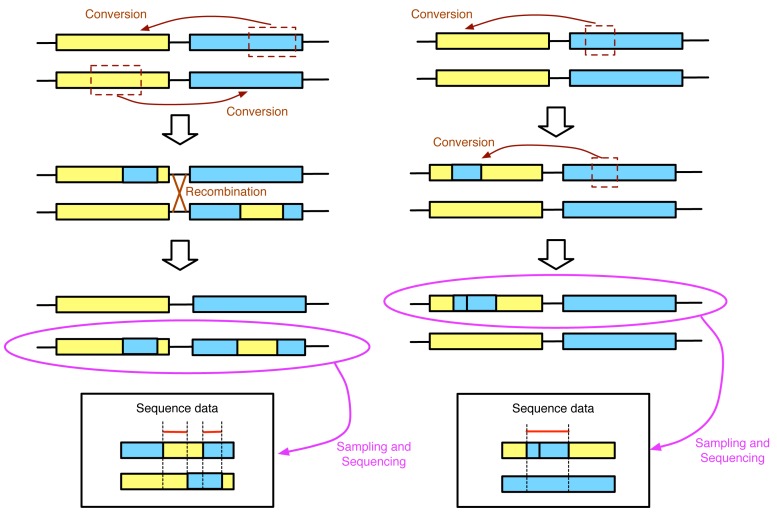
Illustration of the effect of multiple gene conversions on the performance of GENECONV. See text for details.

As was demonstrated by our recent simulation work [75], it is not appropriate to use GENECONV to infer the actual tract length. There is no doubt that the regions identified by GENECONV are strong candidate regions that have undergone recent gene conversion. However, the identified region is not necessarily to correspond to the region that was really transferred by a single gene conversion event. Accordingly, the result of GENECONV is sometimes misinterpreted as if the output (a list of candidate converted tracts) reflects the distribution of the tract length of gene conversion (*i.e.*, Ref. [76]). This effect is easily demonstrated by simple illustrations in Figure 4. In the left panel, two conversions in the opposite directions share a part of the tract. If GENECONV is applied to the sequence data in the box, it will likely identify two regions (with red lines in Figure 4) with lengths much shorter than the real converted tract lengths. In contrast, the two overlapping conversion events in the same direction result in a large region identified by GENECONV. The real situation should be much more complicated with a number of overlapping recurrent gene conversion events, indicating that the empirical approach would be the only reliable means to investigate the tract length of gene conversion. It should be noted that there are several algorithms for detecting gene conversion tracts [77–81], and we here treated GENECONV as a representative of them because they share the basic idea. Thus, using natural variation in DNA sequences is not very suitable to investigate the tract length of gene conversion.

However, there are special cases where we can obtain high quality of data. One example is disease genes in humans. There are a number of human diseases caused by gene conversion, as reviewed by Chen *et al.* [41] in this issue (see also [3]). In many cases, diseases are caused by gene conversion that transfer a mutation from a pseudogenized duplicate to the functional copy, resulting in missense mutation [82–84], nonsense mutation [85], frameshift [86,87], change of splicing site [88]. These conversions occur between highly similar sequences (more than 90%, see [3]), which seems to be slightly higher than that for yeast and rodent. When the sequences of the functional and pseudogenized copies are known, the positions of causal mutations can be identified. In such a case, there are some interesting follow-up studies that a region encompassing the causal mutations is resequenced or genotyped for a number of patients. The ML method can be directly applied to such a data set to estimate the average tract length with one condition, that is, each of the detected gene conversions was created by a single gene conversion event. This assumption may be reasonable for serious diseases, for which the causal mutation can not increase in frequency in the human population. If so, almost all mutations should be eliminated from the population in a very short time, during which it is quite unlikely that another gene conversion occurs in the focal region. As a consequence, the identified gene conversion tracts in sequence data are most likely created by a single independent gene conversion. It should be noted that, gene conversions under our analyses here are those occurred in meiosis, while the data in Table 1 are for mitosis.

**Figure 5:**
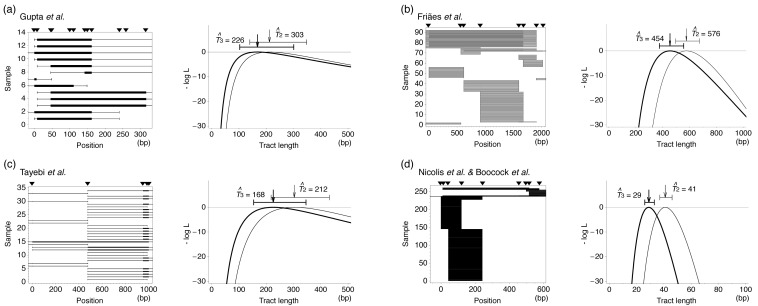
Application of the ML method to the four data sets in Table 2. See the legend of Figure 3 for details.

In Table 2, we summarize four genotyping data sets from human disease genes, for which our assumption of independent gene conversion should likely hold because the frequencies of patients are extremely low. The ML function (8) was applied to these four data sets and the results are summarized in Figure 5. The ML estimates of *T* are quite similar to those of the empirical approach for yeast and rodent.

**Table 2.** Summary of the data of human disease genes used for the ML analysis.

**Data Set**	**Disease**	**Recipient Gene**^a^	**Analyzed Region**	**Sequence Identity**	*n* **(Sample Size)**	**Frequency of Patients**^b^
Gupta *et al.* [89,90]	von Willebrand disease types 2M & 3	*vWF* (12pl3.3/22qll.22—qll.23)	intron 27 and exon 28	97%	13	< 1/500 (type 2M) 1/500,000 (type 3)
Friães *et al.* [91]	congenital adrenal hyperplasia	*CYP21A2* (6p21.3, 30 kb)	exons 1 — 10 and flanking regions	96—98%	92	1/15,500—1/280
Tayebi *etal.* [92]	Gauchar disease	*GBA* (lq21, 16 kb)	exons 3—11	96%	34	1/1,000,000 —1/850
Nicolis *et. al.* [93]	Shwachman- Diamond syndrome	*SBDS* (7qll, 305 kb)	exons 1—5 and junction of exon/intron	97%	25	1/100,000
Boocock *et. al.* [88]					235	

a The chromosomal positions of the donor and recipient genes are shown in the parentheses if they are located on different chromosomes. If they are on the same chromosome, the position of the recipient gene and the distance between the two genes are shown. Note that all donor genes are pseudogenes of each functional gene.

b This frequency includes patients that are not caused by gene conversion.

## 4. Conclusions and Perspectives

As well as other mutational mechanisms, interlocus gene conversion could create changes in DNA sequences, which will potentially cause increase or decrease of the fitness of the host individual. When the fitness impact is strong, the conversion should be immediately selected for or against. To understand the role gene conversion plays in organism's life and evolution, it is crucial to know the rate and tract length of gene conversion.

In this review, we first overviewed the empirical and evolutionary approaches to estimate the rate of gene conversion between duplicated regions. The empirical approach generally involves mutation accumulation studies. Most studies use strains or cell lines, to which donor and/or recipient genes are transferred. This transgenic system, which is well established in yeast and rodent, is flexible enough to explore the rate under variable conditions. It has been demonstrated that the rate is determined by many factors including the genomic location and nucleotide identity between duplicates. Evolutionary approaches may be suitable to estimate the rate for non-model species, because they can be applied when DNA sequence data are available. Estimates based on polymorphism (SNP) data are roughly in agreement with those of the empirical approach.

Inferring tract lengths is more complicated in both of the two approaches. The empirical approach uses the transgenic system for estimating the rate with a modification; A number of markers are distributed in the donor (or recipient) sequences which make it possible to trap conversion tracts. As this system just allows one to identify the initiation and termination positions of the tract between markers, it is difficult to know the actual length. Therefore, we here used a simple ML method to estimate the mean tract length. The method well estimated the mean lengths with relatively narrow confidence intervals for many data sets, indicating the importance of statistical analysis of empirical data. Most estimates range from 50 to several hundred bp.

In contrast, evolutionary data are not very informative for the tract length mostly because evolutionary data accumulate a number of footprints of historical gene conversions that potentially overlap with one another. Exceptions include genotyping data of human disease genes, at which gene conversion causes serious disease when it transfers a deleterious mutation from a pseudogenized duplicate. With this condition, if we have a sample of patients that are not genetically related, most of the detected gene conversions in the gene should be very young and independent. Our ML method was successfully applied to such data.

The rate of gene conversion we considered in this review is a per-site rate, that is, the rate at which a particular site is involved in a gene conversion event. This rate is different from the rate that a gene conversion event occurs. Under the model we introduced above, the former is denoted by *c* and the latter is *g*. *g* can be estimated if we know the average tract length, *T*, because *g* is simply given by *c*/*T*. According our ML analysis, *T* would be on the order of 10^1∼2^ bp, so that the initiation rate of gene conversion per site would be one or two orders of magnitude lower than *c*.

Thus, our knowledge on the rate and tract length of interlocus gene conversion is growing. However, data are still limited to several model systems. Because the rate should be highly variable across the genome, to understand the genomic landscape of gene conversion should be one of the future directions. It has been thought that many biological features are shared by gene conversion and recombination, and the recombination rate is also highly variable across the genome. There are a number of hotspots of recombination in genomes [73,94–97], and some of them would also be associated with interlocus gene conversion [98–100] although the amount of information is still limited. To fully understand the mechanisms behind the great variability of the gene conversion and recombination rates, we need much more data than currently available, part of which may be obtained by taking advantage of next-geneartion sequencing.
